# Discrimination Between Commercial Tomato Juices from Non-Concentrate and Concentrate Based on Their Volatile Profiles

**DOI:** 10.3390/foods14172993

**Published:** 2025-08-27

**Authors:** Yoko Iijima, Katsutoshi Saisho, Taiki Maeoka

**Affiliations:** Department of Applied Chemistry, Kogakuin University, Tokyo 192-0015, Japan; s218038@g.kogakuin.jp (K.S.); s219073@g.kogakuin.jp (T.M.)

**Keywords:** tomato juice, tomato concentrate, VOC, flavor quality, HS-SPME, OPLS-DA, Strecker aldehydes

## Abstract

Commercial fruit juices are categorized into juice not from concentrate (JNFC) and juice from concentrate (JFC). Tomato juice is one of the most popular vegetable juices, and its aroma is an important factor in evaluating its quality. However, differences in the aroma characteristics of JNFC and JFC tomato juices have not been clearly identified. This study aimed to investigate the volatile organic compounds (VOCs) involved in distinguishing between JNFC and JFC using commercially available tomato juices. Furthermore, the effect of concentration on the VOC composition was evaluated using different procedures. Twenty-three commercial tomato juices were prepared for analysis of VOCs using headspace solid phase microextraction-gas chromatography mass spectrometry (HS-SPME-GC-MS). Principal component analysis (PCA) and orthogonal partial least squares discriminant analysis (OPLS-DA) were used to discriminate the samples into JNFC and JFC groups. JNFC contained 43 VOCs, which was more than twice that contained in JFC, and the quantitative variation was larger in JNFC than in JFC. In particular, the JNFC group contained significantly more alcohol and phenol compounds. On the other hand, the JFC group contained more formyl pyrrole and Strecker aldehydes. Additional GC-MS/olfactometry (GC-MS/O) and odor active value analyses indicated that (*Z*)-3-hexenol and 3-methylbutanal were the best VOCs to distinguish between the JNFC and JFC groups. Furthermore, different concentration procedures, including heating concentration (HC), decompression concentration (DC), and freeze drying (FD), were performed, and the corresponding VOCs were compared. HC and DC reduced the levels of most of the compounds to the levels seen in commercial JFC. These results indicate that the concentration procedure is an important processing stage, in addition to the break process, that determines the quality of tomato juice.

## 1. Introduction

Tomato juice is one of the most popular vegetable juices around the world. It has the unique flavor of tomato with various functionalities related to health promotion, and as such is expected to be a valuable product for consumers [1,2,3]. Tomato juice is processed and then canned, packed, or bottled for purchase from stores for use in cooking and as a drink.

In Japan, commercial tomato juice is strictly defined by the Japanese Agricultural Standards as follows: (1) juice extracted from crushed and squeezed or pureed tomato from which the skin and seeds were removed (hereinafter referred to as squeezed tomato juice), with or without salt as the only additive; (2) diluted juice returned to squeezed juice from concentrated tomato extract, with or without salt as the only additive. Therefore, only salt is allowed as an additive for juice sold with the label “tomato juice”, and other additives, such as flavorings and colorants, are not permitted. This means that the flavor of commercial tomato juice is derived only from the tomato materials, with compounds generated or lost during processing. In general, juice not from concentrate (JNFC) is expected to have the flavor of fresh tomatoes, but it is only produced in limited quantities during the summer. It has been reported that the varieties of tomato, break conditions, i.e., hot break (incubation at 85 to 90 °C) or cold break (incubation at 60 to 75 °C), and pasteurization affect tomato juice quality [4,5]. We previously conducted an integrated analysis of 15 commercial tomato JNFC samples produced in Japan for aroma component analysis by gas chromatography mass spectrometry (GC-MS) and sensory evaluation [6]. As a result, the aroma characteristics and compositional profiles were determined to differ significantly due to the tomato cultivars used and the production methods employed by each manufacturer. However, previous studies on the flavor quality of tomato juice have focused only on tomato varieties or the effect of heating during production [7,8,9].

Other than JNFC, juice from concentrate (JFC) is more available at a lower cost all year round. The advantages of tomato juice concentrate include its ease of storage and transport, as well as its ability to maintain its flavor and texture regardless of the season or harvest time. Furthermore, it can be adjusted for various uses and diluted to the desired concentration. In the processing of tomato JFC, the additional processes of concentration and reduction are considered to be involved in the formation of flavor. However, there are few studies that have focused on and summarized the differences in the aroma characteristics of commercial JNFC and JFC.

Evaporation at atmospheric or decompressed pressure is most useful to concentrate juice. However, evaporation under atmospheric conditions cannot avoid the thermal effect, which leads to the production of an off-flavor derived from the cooked condition [8,9]. For evaporation under decompressed conditions, we do not need to consider the thermal effect; however, evaporation can cause a loss of flavor, especially the loss of highly volatile organic compounds (VOCs). In addition to evaporation, freeze condensation is also effective to reduce processing effects and is reported to maintain a fresh flavor [10,11]. In freeze condensation, water in the juice is removed as ice by solid–liquid distribution, with low energy needed for concentration. In addition, concentration with a membrane, such as reverse osmosis (RO), has also been applied for various juices recently [12,13,14]. This process does not require energy for concentration, and it is possible to keep the original flavor, although achieving a high concentration is difficult. It is not typically required that commercially available JFCs are labeled to identify the concentration methods for consumers, which makes it difficult to distinguish and characterize the flavors of JNFC and JFC with respect to the concentration method.

In this study, we analyzed the VOCs of various commercially available tomato JNFCs and JFCs and compared their profiles to identify common marker compounds that distinguish them across varieties and manufacturers. Next, several concentration and reduction processes were conducted using model juices, and the compositional changes of VOCs in them were estimated. In addition, we investigated quantitative trends in aroma-contributing components through various concentration procedures and estimated their impact on juice flavor. These findings should lead not only to an understanding of flavor profiles based on VOCs, but also to the development of ideal models to verify the authenticity of juices and manufacturing techniques.

## 2. Materials and Methods

### 2.1. Samples

Twenty-three commercial pure tomato juices were purchased from local markets in Japan (Table 1), all of which were made from pure tomato without salt or additives. Detailed product names and manufacturers’ names are shown in Table S1. Thirteen of these juices were JNFC, while 10 juices were from JFC. After opening, the juices were immediately transferred to 50 mL plastic bottles and frozen at −80 °C until use. Four manufacturers provided both JFC and JNFC juices; therefore, these samples were used for sensory analysis and comparison. Among these, sample JNFC_1 (Kagome tomato juice premium 2021, Kagome Co. Tochigi, Japan) and sample JFC_2 (Tomato juice 2021, Nippon Del Monte Co., Gunma, Japan), representatives of JNFC and JFC, respectively, were used for GC-MS/olfactometry (GC-MS/O) analysis. Sample JNFC_1 was used as a model for analysis of concentration/recovery and for heating.

### 2.2. Reagents and Standards

Analytical grade calcium chloride, sodium chloride, dichloromethane, and ethanol were purchased from FUJIFILM Wako Pure Chemical Corp. (Osaka, Japan). An alkane standard mix (C4-C30) was obtained from Hayashi Pure Chemical Ind., Ltd. (Osaka, Japan). Analytical grade standard VOCs were purchased from Tokyo Chemical Industry Co., Ltd. (Tokyo, Japan) or Sigma-Aldrich Japan (Tokyo, Japan).

### 2.3. Measurement of the Basic Properties of the Juices

The basic properties of the juices were measured, such as the viscosity, acidity, and total soluble solid content (degrees Brix). The viscosity of each juice was measured using a Rapid Visco Analyzer (RVA4500, PerkinElmer Japan G.K., Kanagawa, Japan). Twenty-five grams of each tomato juice was weighed into an aluminum container dedicated to the RVA4500 apparatus. This aluminum container was coated with a protective agent provided by the manufacturer to avoid the influence of pH. The paddle was rotated at 160 rpm at 25 °C. Viscosity was measured constantly during an 8 min period, and the stable value at the end of measurement was defined as the viscosity of each tomato juice. Brix and acidity were measured using a Brix-Acidity meter based on electrical conductivity (PAL-BX/ACID F5, ATAGO Co., Ltd., Tokyo, Japan) [15,16]. According to the manufacturer’s protocol, the Brix of each tomato juice was analyzed using 0.4 mL samples. Acidity was measured using 0.4 mL of a 50-fold dilution of each sample in according to the manufacturer’s protocol. The acidity was expressed as citric acid equivalents.

### 2.4. Sensory Evaluation

Flavor differences between JFC and JNFC were confirmed by sensory evaluation. The four pairs of JFC and JNFC juices from four different manufacturers were used. Sensory evaluation was performed by paired comparison test based on ISO 5495:2005 [17]. Eighteen panelists (5 women and 13 men, ages: 21–24) were recruited from among the students of department of applied chemistry at Kogakuin University and trained by tasting various tomato juices preliminarily prepared before the actual test. Each pair of tomato juices (JFC and JNFC made by the same company) was prepared and tested. Each tomato juice (35 mL) was poured into a 3 oz white paper cup, covered with aluminum foil, and then provided to the panelists. The following specific terms were chosen for the test: “fresh tomato-like aroma”, “fresh tomato-like flavor”, “cooked tomato-like aroma”, and “cooked tomato-like flavor”.

Here, the “aroma” strength was evaluated by smelling tomato juices before tasting. The “flavor” strength was evaluated after tasting. In one test, the panelist was allowed to repeatedly compare samples. Although no time limit was set for the test, all panelists completed their evaluations within 3 min. This sensory study was approved by the Research Ethics Committee of Kogakuin University (File number: 2021-A17, 11 January 2022).

### 2.5. VOC Extraction by Headspace Gas/Solid-Phase Microextraction (HS-SPME)

VOC extraction was performed according to our previous report [6]. Each tomato juice (2 mL), 1.5 g of calcium chloride, and 2 mL of ultrapure water was stirred in a 20 mL vial. Ten microliters of an aqueous 1-ethylcyclohexanol solution (1.16 μg mL^−1^) as an internal standard was added to this vial, followed by sonication at 40 kHz for 5 min (ASU-10, AS ONE, Osaka, Japan). HS-SPME was adopted to extract the VOCs from the tomato juices. An MPS2-xt autosampler (GERSTEL GmbH & Co.KG, Mülheim (Ruhr), Germany) was used for extraction and injection of the VOCs into the GC-MS apparatus. The headspace gases of the tomato juice samples were saturated by incubation of the analyzed sample at 50 °C for 10 min at 250 rpm, and the SPME fiber (DVB/CAR/PDMS 50/30 (2 cm), Sigma-Aldrich) was exposed to the headspace gases for 20 min to adsorb the VOCs. Desorption of the VOCs from the SPME fiber was performed by direct introduction into the injection port of the GC-MS apparatus, maintained at 250 °C for 5 min. Analysis of each sample was performed in triplicate. The detailed GC-MS conditions are shown in 2.9.

### 2.6. Confirmation of the Aroma Characteristics of VOCs by GC-MS/Olfactometry (GC-MS/O) Analysis

To confirm the aroma characteristics of detected VOCs, GC-MS/O analysis was performed using aroma extracts from the tomato juices. Samples JNFC_1 and JFC_2, respective representatives of the JNFCs and JFCs, were used for the GC-MS/O analysis. Highly volatile potent aroma compounds were detected from the HS-SPME extract prepared in the previous section. However, the aroma characteristics of lower volatile compounds could not be detected from the HS-SPME samples. Therefore, these were detected using solvent extracts from tomato juices for samples JNFC_1 and JFC_2, after two dilutions with ultrapure water. Sodium chloride (40 g) was added to 200 mL of each diluted sample and extracted with dichloromethane (100 mL) for 1 h at room temperature. After centrifugation (300× *g*, 5 min), the lower layer was collected, and the volatile compounds were separated by solvent-assisted flavor evaporation (SAFE) at a pressure of <3.0 × 10^−3^ Pa. The volatile fraction was concentrated in a rotary evaporator at slightly reduced pressure (550 Torr, 35 °C) with a gentle flow of N_2_ gas to 0.5 mL [18]. The obtained aroma concentrates were confirmed to retain the aroma of the original tomato juices by smelling the mouillette into which they were dropped (2 µL). The obtained aroma concentrates were analyzed using GC-MS/O. The same gas GC-MS system used above was employed, but the column outlet was connected to a mass spectrometer and a Gerstel ODP-3 olfactory detection port (GERSTEL GmbH & Co.KG). The detailed GC-MS conditions are shown in Section 2.9.

### 2.7. Preparation of Model Juice Samples by Concentration and Recovery

Three concentration and recovery samples were prepared from JNFC_1 in 2024. Concentration by heating was performed at 80 °C with stirring at 600 rpm until the tomato juice was concentrated to 40 g. The same juice was subjected to vacuum evaporation and to two kinds of freeze drying using 80 g samples. The vacuum evaporation conditions were set to 100 Torr, and the tomato juice was evaporated to 40 g under 40 °C with rotation at 70 rpm. Freeze drying was performed at 30 Pa and −50 °C using an FDU-1200 freeze dryer (Eyla, Tokyo, Japan). Each flask (100 mL) containing 80 g of frozen tomato juice was covered with an aluminum foil sheet with pinholes and stored in the freeze dryer at 25 °C. The samples were concentrated to 10 g (87.5% loss of water, FD_87.5) or 40 g (50% loss of water, FD_50). All concentrated tomato juices were restored to their original weights by adding water and then used for aroma analysis with GC-MS. The sample conditions for analysis were the same as those described in a previous section (Section 2.5). Analyses for all samples under each condition were performed in triplicate.

### 2.8. Stability of VOCs in the Sealed Tomato Juice While Heating

To measure the stability of the main aroma compounds, tomato juice sample JNFC_1 was transferred to sealed bottles and heated in boiling water for 30, 60, 180, and 300 min. After heating for each period, the samples were immediately cooled on ice and gently shaken to return the separated volatiles in the headspace volume to the liquid. We transferred 2 mL of each sample to a 20 mL vial and analyzed the VOCs under the same HS-SPME/GC-MS conditions as described in Section 2.5 and Section 2.9. Samples were prepared in triplicate.

### 2.9. GC-MS Conditions

GC-MS analysis was performed using a gas chromatograph (7890B GC, Agilent Technologies, Santa Clara, CA, USA) coupled to a mass spectrometer (5977A, Agilent Technologies). The volatiles were eluted using a fused silica capillary column (DB-WAX UI, Agilent J&W Scientific, Agilent Technologies; 20 m × 0.18 mm i.d., 0.3 μm film thickness). The oven temperature was set at 40 °C with a 3 min hold and raised to 240 °C at a rate of 5 °C min^−1^, then held at 240 °C for 17 min. The total running time was 60 min. The injection port was set at 240 °C. Helium was used as the carrier gas at a flow rate of 1 mL min^−1^. Mass spectra were obtained under the following conditions: ionization voltage, 70 eV (EI); ion source temperature, 230 °C; quadrupole temperature, 150 °C; mass range, *m*/*z* 33–350.

A fused silica capillary column (DB-WAX UI, Agilent J&W Scientific; 60 m × 0.25 mm i.d., 0.25 μm film thickness) was used for the GC-MS/O analysis. Helium was used as the carrier gas at a flow rate of 1.8 mL min^−1^ under constant pressure mode (208 kPa). The split ratio between the MS and the sniffing port was set at 1:1. The oven temperature was set at 40 °C with a 5 min hold and raised to 240 °C at a rate of 4 °C min^−1^, then held at 240 °C for 30 min. The HS-SPME fibers for highly volatile VOCs were injected into the injection port in splitless mode. For lower volatiles, 3 μL of aroma concentrates were supplied to the injection port in splitless mode. The injection volume was set after calculation using the Agilent Technology GC calculators that the GC-MS instrument was equipped with. Among the detected compounds, those that could be confirmed by two of the three panelists were defined as aroma-active compounds.

### 2.10. Compound Identification and Quantification

Each compound was identified by comparison of the Retention Index (RI) and mass spectrum with each standard compound we obtained. For unavailable standards, annotation was performed using Aroma Office (ver. 7, Nishikawa Keisoku Co., Ltd., Tokyo, Japan) with NIST library ver. 17 (Agilent Technologies) based on the retention index and mass spectra literature. Quantification of each compound was performed by spiking the tomato juice samples with an authentic standard dissolved in ethanol. The peak ratio of the spiked authentic standard to the internal standard peak was calculated for quantification.

### 2.11. Data Processing, Multivariate Analysis, and Statistical Analysis

Data processing and multivariate analysis were performed according to our previous report [6]. VOC data were collected in triplicate per sample, and the average values were used for multivariate analysis to clarify the differences between samples. Principal component analysis (PCA) and orthogonal partial least squares discriminant analysis (OPLS-DA) were performed using Metaboanalyst 5.0 [19].

Statistical differences were evaluated by one-way analysis of variance (ANOVA) followed by Tukey’s honestly significant difference (HSD) post hoc test using BellCurve for Excel (Social Survey Research Information Co., Tokyo, Japan). The Brunner–Munzel test was performed to evaluate differences between the NFC and JFC groups for each VOC, as the two groups were not equally distributed. The significance of the sensory evaluation results was determined using a one-sided test based on binomial distribution.

## 3. Results and Discussion

### 3.1. Differences in Flavor Characteristics Between JFC and JNFC, as Determined by Sensory Evaluation

Differences in the flavor characteristics between the JFC and JNFC samples were determined by sensory evaluation. The basic chemical information for JNFC (13 samples) and JFC (10 samples) is given i\\n Table 1. Detailed product information for each sample is presented in Table S1. The Brix and viscosity values were within 4.4–8.0° and 72–188 mPa·s, respectively. The correlation coefficient between the Brix and viscosity values was 0.70. Among the samples, JNFC_7, JNFC_8, and JNFC 11 of the JNFC samples showed higher values. On the other hand, the acidity of all the samples was similar and within 0.48–0.98 *w*/*v* %. Four tomato juice manufacturers (A–D) produced both JNFC and JFC samples; therefore, the sample pairs from each manufacturer were compared and evaluated for fresh tomato and cooked tomato characteristics by paired comparison test (Table 2). The strengths of each characteristic were evaluated for aroma by smelling and flavor by tasting. The JFC samples from all manufacturers were scored as having a more cooked aroma and flavor than the JNFC samples. On the other hand, the JNFC samples were considered to have more fresh tomato aroma and flavor. In particular, those from companies B and D were significantly distinct in terms of both aroma and flavor. The stronger cooked aroma/flavor of the JFC samples was considered to be due to the effects of heat processing during concentration of the tomato juices.

### 3.2. Differentiation of Tomato Juices Based on VOC Composition

**Figure 1:**
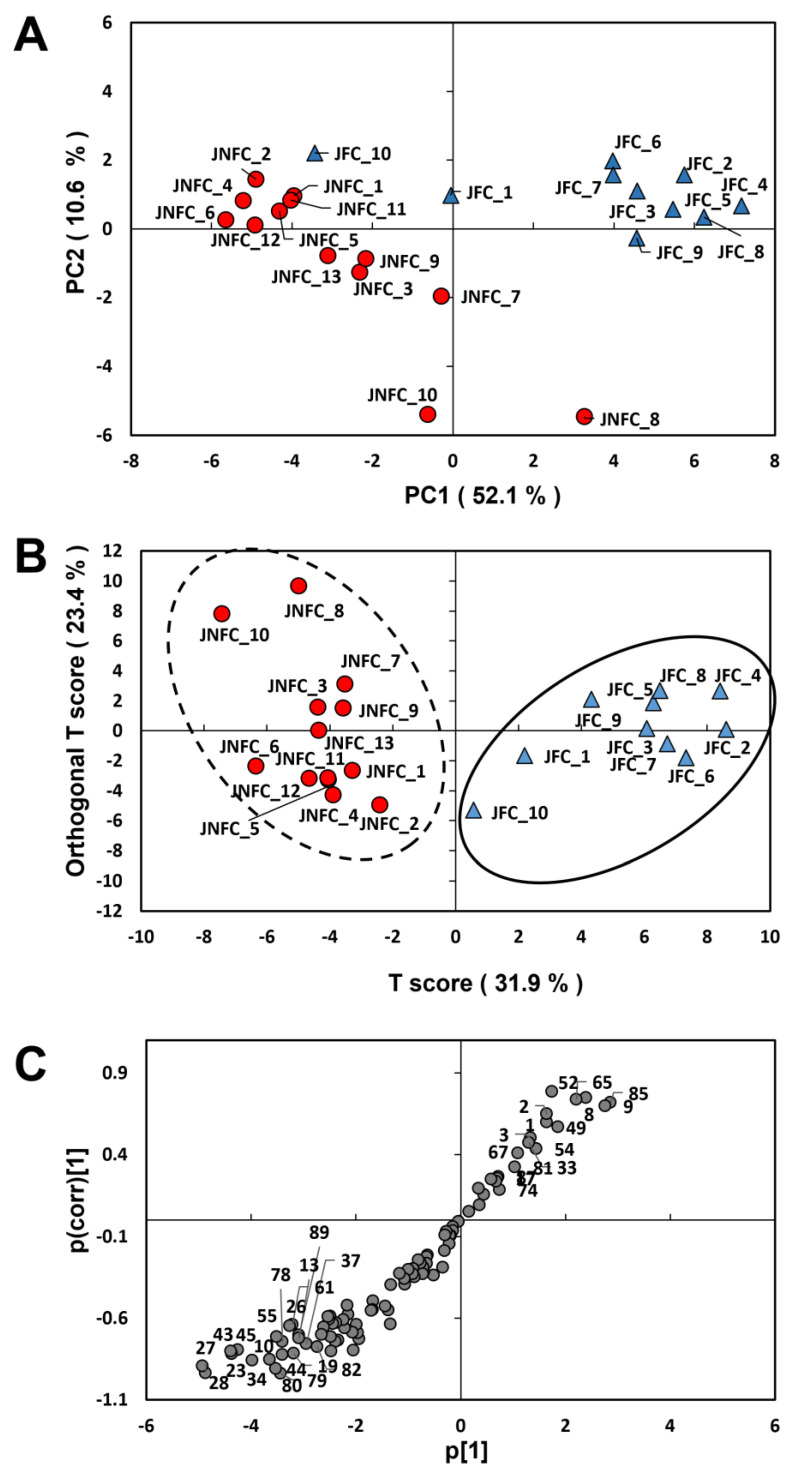
Multivariate analysis based on the VOC profiles of the tomato juices. (**A**) Score plot based on PCA analysis. Each number indicates samples in Table 1 (JNFC 1-13 and JFC 1-10). (**B**) Score plot based on OPLS-DA. Each number indicates the same as (**A**). (**C**) S-plot based on OPLS-DA. Each number indicates the detected VOCs, which are the same as those in Table 3.

The viscosities of the tomato juices differed depending on the samples (70–188 mPa·s) (Table 1). To avoid the influence of viscosity on aroma release, all sample juices were diluted twice with water and then used for volatile analysis. The final viscosities of all the samples were confirmed to be within the range of 25 to 30 mPa·s. Volatile analysis of the tomato juices was performed using HS-SPME-GC-MS. In total, 84 compounds were identified by comparing their mass spectra and RI values with standard compounds. The other 10 compounds were annotated using the Aroma Office software 8.0 with the NIST 17 library based on the literature (Table 3). These 94 compounds were used for the subsequent multivariate analysis. The specific *m*/*z* of each compound was determined, and the abundance relative to the specific *m*/*z* of the internal standard (*m*/*z* 99 in mass spectra of 2-ethylcyclohexanol) was calculated and transformed to a log10 value. The original data from the multivariate analysis are shown in Table S2. Figure 1A shows a score plot by PCA based on the VOC composition. Although sample information is not typically considered in PCA, most of the samples clustered separately into the JFC or JNFC group on the PC1 axis (52.1%). This indicates that most tomato juices could be identified as being from concentrate or not based on their volatile profile. Among the samples, JFC_10 and JFC_1 in the JFC group and JNFC_8 in JNFC group clustered differently than the other samples, which suggests that these samples were made by specific processing procedures that differed from the other samples.

**Figure 2:**
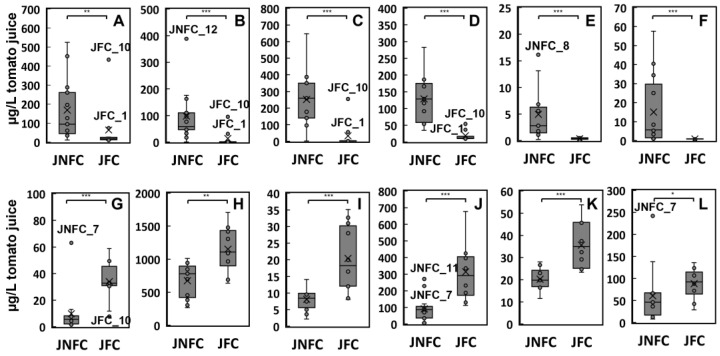
Quantitative differences in the main VOCs that contribute to discrimination between the JNFC and JFC samples. (**A**) Hexanal, (**B**) hexanol, (**C**) (*Z*)-3-hexenol, (**D**) 2-phenylethanol, (**E**) 2-methoxyphenol, (**F**) eugenol, (**G**) formyl pyrrole, (**H**) dimethyl sulfide, (**I**) phenylacetaldehyde, (**J**) 3-methylbutanal, (**K**) methional, and (**L**) furfural. The Brunner–Munzel test was performed to evaluate differences between the JNFC and JFC groups: ***, *p* < 0.001, **, *p* < 0.01, *, and *p* < 0.05.

Next, OPLS-DA was performed to determine the factors that distinguish the JFC and JNFC groups (Figure 1B). The generated OPLS-DA model yielded good discrimination of the JFC and JNFC samples, with an R^2^Y value (interpretation rate of the objective variable by the explanatory variables) of 0.707 and a Q^2^ value (model predictive ability) of 0.663. The S plot clearly discriminated between the VOCs abundant in the JFC versus the JNFC group (Figure 1C). The variable importance in projection (VIP) scores of each compound for separation between the JFC and JNFC flavors are summarized in Table 3. The VIP scores indicate the importance of each explanatory variable (VOC) that contributes to the projection of the OPLS for objective variables (juice samples). VOCs with higher VIP scores are more likely to make a significant contribution to discrimination between the samples. In addition, a volcano plot was generated for the VOCs, and fold changes between JNFC versus JFC samples and their significance differences (*p*-values) are also shown together in Table 3. These results indicated that the JNFC samples contained more VOCs than the JFC samples. Among all the VOCs, 43 were detected more than twice in the JNFC samples with a significance of *p* < 0.05. In particular, alcohols and phenols such as eugenol (peak 89), hexanol (peak 43), (*Z*)-3-hexenol (peak 45), and 2-phenylethanol (peak 80) were more than five times more abundant in JNFC than in JFC (Figure 2). In many previous reports on tomato fruit aroma, it has been reported that C_6_-compounds such as hexanal, (*Z*)-3-hexenal, hexanol, and (*Z*)-3-hexenol, which have a green aroma, are important factors in the flavor freshness of tomatoes [20,21]. Hexanal and (*Z*)-3-hexenal are much more abundant than hexanol and (*Z*)-3-hexenol in tomato fruit [22]; however, they are depleted more than hexanol and (*Z*)-3-hexenol by alcohol dehydrogenase or by release during processing [8,23]. We have reported that hexanol and (*Z*)-3-hexenol are significantly correlated to the fresh aroma in commercial tomato juices by cross analysis of VOC composition and sensory evaluation [6]. Therefore, their loss from the JFC group during concentration suggests that this is responsible for the difference in the aroma characteristics between the JNFC and JFC groups. However, their variation in the JNFC samples was much greater than in the JFC samples (Figure 2). For example, the content ranges of (*Z*)-3-hexenol and 2-phenylethanol were 2–648 μg L^−1^ and 35–283 μg L^−1^ in the JNFC group, respectively. On the other hand, their variation in the JFC samples was low, with smaller amounts, although high amounts were detected in some outlier samples (JFC_1 and JFC_10). The high contents of alcohol compounds in these two samples may be responsible for their isolation from other JFC samples in the PCA (Figure 1A). Sample JFC_1 was produced from concentrate derived using an RO membrane, which suggests that this method resulted in the fresh aroma being maintained. JFC samples contained more aldehydes such as formyl pyrrole (peak 85), 2- and 3-methylbutanal (peak 8 and 9), phenylacetaldehyde (peak 65), methional (peak 52), acetaldehyde (peak 1), and furfural (peak 54) (Table 3). The VOC with the greatest relative difference that was more abundant in the JFC group was formyl pyrrole (peak 85), which was detected in 3.45-fold greater amounts than in the JNFC group (Table 3). This was followed by Strecker aldehydes, 3-methylbutanal (peak 9), phenylacetaldehyde (peak 65), 2-methylbutanal (peak 8), and methional (peak 52), attributed to amino acids. These are reported to be produced by heat treatment in a variety of beverages, as well as tomato products [9,24,25]. This indicates that the thermal conditions for concentration in the processing of the JFC group influenced the generation of these aldehydes.

### 3.3. Odor Activity of Aroma Active Compounds

VOCs that contribute to the separation of the JNFC and JFC groups were identified; however, their contribution to aroma activity was unclear. Therefore, we focused on aroma-active VOCs and investigated their contents in each sample. First, we confirmed the aroma characteristics of VOCs in tomato juices by GC-MS/O with HS-SPME. However, a sufficiently strong aroma could not be detected for some VOCs at the sniffing port on the GC. Therefore, aroma concentrate prepared by solvent (dichloromethane) extraction and SAFE from representative JFC and JNFC tomato juices (JNFC_1 and JFC_2) were used for GC-MS/O analysis to confirm the aroma-active compounds.

The odor descriptions and odor activity values (OAVs) calculated based on contents and each threshold level are given in Table 4. In total, 22 odor-active compounds were detected in the samples. They included most of the marker compounds that contributed to distinguishing between the JFC and JNFC groups in Figure 1 and Figure 2. In both sample groups, the OAVs of dimethyl sulfide, 3-methylbutanal, β-damascenone, and β-ionone showed high levels. Dhuey et al. reported that sulfur compounds such as dimethyl sulfide, dimethyl disulfide, and dimethyl trisulfide increased after retort processing of tomato [26]. In particular, dimethyl sulfide was generated from S-methyl methionine and was significantly increased, approximately 20-fold, after processing at 121 °C for 30 min, and its content remained at high levels for 50 days. Therefore, the results of the high dimethyl sulfide content in all the tomato juices suggested that this was due to heat treatment for pasteurization, a process common to all samples. 3-Methylbutanal, which has a malty cooked aroma, had high OAVs in both the JNFC and JFC groups, although the value was greater in the JFC group. The formation of 3-methylbutanal occurs through two pathways. One is by Strecker degradation from leucine, based on the thermal conditions [27]. The other is from a biosynthetic pathway by branched-chain amino acid aminotransferase and α-ketoacid decarboxylase in tomato fruit [28]. Therefore, abundant 3-methylbutanal was detected even in freshly ripened tomato fruit, as previously reported [29,30]. However, the present results revealed that the JFC group contains relatively higher amounts (Figure 2J), which suggests it was generated by Strecker degradation due to a thermal effect during concentration. In addition, methional, with a strong boiled potato aroma, was also detected above the odor threshold as another Strecker aldehyde.

The OAVs for hexanal, hexanol, and (*Z*)-3-hexenol indicated that (*Z*)-3-hexenol is most responsible for the fresh green note of the tomato juices, as shown in Table 4. The smaller amount of (*Z*)-3-hexenol in the JFC samples results in the less fresh aroma and flavor of the JFC samples compared with the JNFC samples, as determined by sensory evaluation (Table 2).

### 3.4. Effect of Concentration Condition on the Aroma Profiles of Reduced Tomato Juices

**Figure 3:**
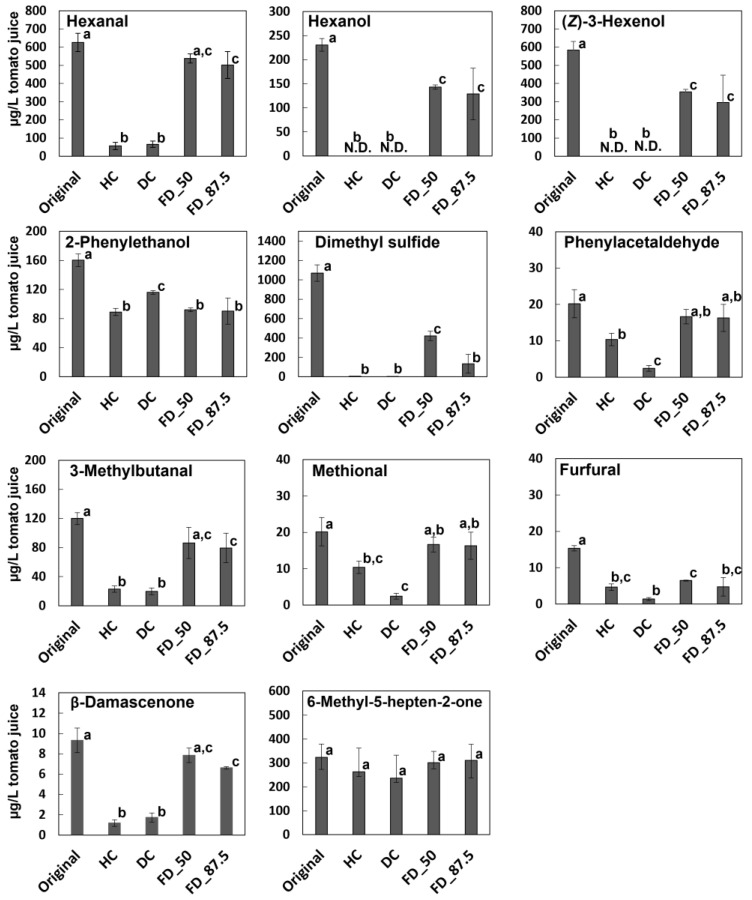
Quantitative comparison of the main aroma-active compounds in the tomato juices. Original, HC, DC, FD_50, and FD_ 87.5 indicate original juices (standard), reduced juice from concentrate by heat, decompression, and freeze drying (50% and 87.5% loss of water), respectively. The data are expressed as the mean ± standard error of the mean (N = 3). Different letters indicate significant differences (*p* < 0.05, Turkey’s HSD test).

The processing of commercial tomato juices in each company is kept secret and not disclosed to the public. Therefore, we investigated the effect of concentration with four model procedures: heat concentration (HC), decompressed concentration (DC), and freeze drying for 50% (FD_50) and 87.5% (FD_87.5) loss of water. A JNFC sample (JNFC_1) was used for the concentration model. After concentration, the samples were diluted with water to the original volume, and VOC analysis was conducted. The differences in the content of the main aroma active compounds due to the concentration method is shown in Figure 3.

**Figure 4:**
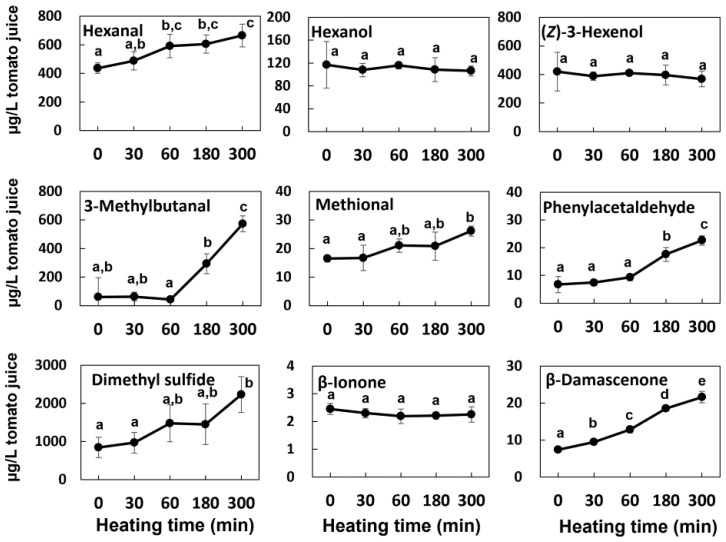
Thermal stability of the main aroma compounds in tomato juice incubated in boiling water. The data are expressed as the mean ± standard error of the mean (N = 3). Different letters indicate significant differences (*p* < 0.05, Turkey’s HSD test).

Furfural was shown as a typical thermal marker compound [33]. All VOCs were contained more abundantly in the original sample than in the concentrated and reduced samples. However, the effect of each concentration process differed according to each VOC structure. Hexanal, hexanol, and (*Z*)-3-hexenol, which are more highly volatile, were almost lost by concentration under heat or reduced pressure. This result was consistent with the JFC samples. To confirm the thermal stabilities of these VOCs in tomato juice, we prepared a juice by heating it for 300 min in a sealed container, and then performed VOC analysis. In Figure 4, all of these VOCs were stable during heating, which indicated that their loss during concentration and reduction occurred by evaporation. On the other hand, freeze drying was effective to reduce the loss of the most aroma-active VOCs, as well as C_6_-compounds, which were retained at levels of more than 50% of the control. Jeyaprakash et al. compared the composition of aroma compounds in tomato powder produced by different drying procedures [34]. Their results were supportive of the present results, in that most VOC contents are sustainable by freeze drying than other methods such as spray drying at more than 40 °C.

2-Phenylethanol and 6-methyl-5-hepten-2-one were not affected by concentration method and remained in high amounts in both the model juices and the JFC samples (Figure 1 and Figure 3). The calculated chemical parameters for volatility, logPow and vapor pressure, were 2.07 and 1.78 mmHg/25 °C for 6-methyl-5-hepten-2-one and 1.57 and 0.0243 mmHg/25 °C for 2-phenylethanol, respectively. Although the vapor pressure for 2-phenylethanol is low, the behaviors with the different concentration methods cannot be explained by basic chemical properties. However, the stable content of 6-methyl-5-hepten-2-one in all of the tomato juices, where its fold change from the JNFCs to JFCs was 1.489 (Table 3), suggests it is consistent with the trends observed for the model concentrations.

Although the loss of most compounds by concentration processing in the model samples can explain the flavor differences between the JNFC and JFC groups, dimethyl sulfide and Strecker aldehydes showed different behaviors between the tomato juices and concentrated and reduced model juices, which were relatively higher in the JFC samples.

Dimethyl sulfide and 3-methylbutanal are highly volatile; therefore, it is reasonable that they are lost during concentration. However, they were increased by heating in a sealed container (Figure 4). Therefore, it is suggested that their original amounts just after the processing of tomato juices could be evaporated by concentration, but they could be regenerated by sterilization after packing of the reduced juices from tomato concentrate.

## 4. Conclusions

Commercial JNFCs and JFCs were compared based on their VOC profiles. Multivariate analysis enabled the identification of VOCs in the JFC and JNFC groups. Some of the VOCs involved in the discrimination of these differences were found to be present in tomato juice at levels above the threshold and were thought to contribute to the sensory differences. In the heat processing of tomato juice, secondary reactions, such as the Maillard reaction and the hydrolysis of glycosides as aroma precursors [35,36], are considered to influence the compositional changes. In this study, we indicated that the concentration process, in addition to heat processing, is an important factor in determining the VOC composition for the flavor quality of tomato juice. There are improvements in concentration technologies used for various fruit juice beverages to maintain a fresh flavor and nutritional profile, such as membrane concentration, freeze concentration, and freeze drying. The development of these technologies could lead to the production of juices that retain the same flavor as the fresh fruit. On the other hand, tomato juice is adopted not only as a beverage, but also as a cooking ingredient; thus, it does not necessarily need to have a fresh aroma in some cases. The production of tomato juice with the flavor desired by consumers could be controlled through these processes. To understand their effects in detail, it should be indispensable to conduct comparative analyses based on systematic implementation by setting conditions in stages for processing conditions, as well as agrotechnical conditions.

## Figures and Tables

**Table 1:** Basic characteristics of the commercial tomato juices used in this study.

Sample No.	Origin of Sample *	Brix (°)	Acidity (*w*/*v* %)	Viscosity (mPa/s)
JNFC_1	JNFC	4.9	0.52	73
JNFC_2	JNFC	5.1	0.84	79
JNFC_3	JNFC	4.6	0.56	84
JNFC_4	JNFC	4.7	0.52	116
JNFC_5	JNFC	5.5	0.71	145
JNFC_6	JNFC	4.7	0.55	83
JNFC_7	JNFC	8.0	0.75	172
JNFC_8	JNFC	7.1	0.74	188
JNFC_9	JNFC	5.6	0.62	72
JNFC_10	JNFC	5.8	0.48	77
JNFC_11	JNFC	8.0	0.81	136
JNFC_12	JNFC	5.1	0.60	129
JNFC_13	JNFC	4.6	0.48	89
JFC_1	JFC	5.4	0.60	84
JFC_2	JFC	4.4	0.51	84
JFC_3	JFC	5.1	0.86	81
JFC_4	JFC	5.8	0.58	133
JFC_5	JFC	5.2	0.98	84
JFC_6	JFC	4.6	0.56	70
JFC_7	JFC	4.9	0.62	106
JFC_8	JFC	5.2	0.60	112
JFC_9	JFC	5.4	0.69	132
JFC_10	JFC	5.2	0.62	73

* JNFC and JFC indicate “not from concentrate” and “from concentrate”, respectively.

**Table 2:** Typical flavor properties of JNFC and JFC, as evaluated by paired comparison test. Juice pairs from the same manufacturer were used. The number of panelists indicating “stronger” for each descriptor is shown.

Sample Pair		A		B		C		D	
		JNFC_1	JFC_1	JNFC_2	JFC_2	JNFC_3	JFC_3	JNFC_6	JFC_4
aroma	fresh	14 ^a^	4	17 ^c^	1	12	6	15 ^b^	3
	cooked	4	14 ^a^	1	17 ^c^	6	12	3	15 ^b^
flavor	fresh	12	6	13 ^b^	5	15 ^b^	3	17 ^c^	1
	cooked	6	12	5	13 ^b^	3	15 ^b^	1	17 ^c^

Significant differences are *p* < 0.05 for “^a^”, *p* < 0.01 for “^b^”, and *p* < 0.001 for “^c^”. Each pair of samples from the same manufacturer is indicated by a capital letter (A–D). Detailed information about the samples is shown in Table S1.

**Table 3:** Identified volatiles from commercial tomato juices and their odor activity and fold changes between JFC and JNFC samples.

Peak No.	RI	Identified Compounds	VIPs for OPLS-DA	Fold Change (JNFC/JFC)	*p*-Value
1	715	acetaldehyde	1.059	0.581	0.021
2	752	dimethyl sulfide	1.151	0.586	0.003
3	803	furan	0.887	0.734	0.049
4	817	acetone	0.168	1.134	0.620
5	870	3-methylfuran	0.618	1.479	0.112
6	893	ethyl acetate	1.043	2.941	0.009
7	901	2-metnylfuran	1.112	3.953	0.002
8	914	2-methylbutanal	1.326	0.400	0.001
9	917	3-methylbutanal	1.235	0.292	0.001
10	940	ethanol	1.510	9.231	0.000
11	952	2-ethylfuran	1.263	3.739	0.000
12	979	pentanal	0.126	1.371	0.844
13	1036	2-butanol	1.279	4.574	0.001
14	1038	toluene	0.116	1.288	0.507
15	1050	2-methyl-3-buten-2-ol	1.132	2.340	0.002
16	1055	ethyl 2-methylbutyrate	0.164	0.456	0.842
17	1071	dimethyl disulfide	0.451	1.058	0.563
18	1085	hexanal	1.073	2.636	0.010
19	1094	2-methyl-2-butenal	1.443	6.958	0.000
20	1113	2-methyl-1-propanol	1.219	2.970	0.001
21	1126	isovaleronitrile	0.092	1.108	0.994
22	1131	*p*-xylene	0.442	0.952	0.402
23	1177	1-penten-3-ol	1.404	6.968	0.000
24	1185	limonene	0.018	1.236	0.975
25	1202	3-methyl-2-butenal	0.584	1.775	0.106
26	1218	(*E*)-2-hexenal	1.144	4.182	0.002
27	1222	2-methylbutanol	1.657	10.857	0.000
28	1223	3-methylbutanol	1.580	8.525	0.000
29	1227	2-pentylfuran	0.635	1.780	0.110
30	1230	(*E*)-β -ocimene	0.970	2.459	0.008
31	1247	2, 4-dimethylphenol	1.025	2.191	0.010
32	1257	2-methyl-1-penten-3-ol	0.529	1.818	0.127
33	1261	methyl propyl sulfide	0.835	0.681	0.050
34	1265	pentanol	1.519	8.120	0.000
35	1274	hexyl acetate	0.435	2.946	0.391
36	1275	4-ethyltoluene *	0.563	1.387	0.130
37	1292	acetoin	1.342	5.222	0.000
38	1301	(*E*)-2-(2-pentenyl)furan *	0.696	1.554	0.090
39	1313	2, 2, 6-trimethylcyclohexanone	0.512	1.339	0.269
40	1316	3, 4-dimethylcyclohexanol *	0.162	1.707	0.478
41	1320	6-methyl-6-hepten-2-one *	0.702	1.805	0.098
42	1343	6-methyl-5-hepten-2-one	0.466	1.489	0.274
43	1367	hexanol	1.422	7.285	0.000
44	1376	(*E*)-3-hexen-1-ol	1.458	5.771	0.000
45	1397	(*Z*)-3-hexen-1-ol	1.448	7.469	0.000
46	1410	2-isobutylthiazole	1.049	2.436	0.012
47	1416	1-(1-methycyclopentyl)-ethanone *	0.924	4.762	0.029
48	1421	perillene *	0.073	1.165	0.987
49	1427	1, 3-di-tert-butylbenzene	1.006	0.485	0.007
50	1451	linalool oxide	0.169	1.132	0.565
51	1457	acetic acid	0.340	0.948	0.555
52	1457	methional	1.388	0.569	0.000
53	1462	1-octen-3-ol	1.242	4.347	0.001
54	1467	-furfural	0.772	0.686	0.046
55	1476	6-methyl-5-hepten-2-ol	1.266	2.096	0.002
56	1522	benzaldehyde	0.332	1.105	0.192
57	1538	2-(methylthio) ethanol	1.303	3.587	0.000
58	1558	linalool	0.980	2.395	0.009
59	1571	(*Z,Z*)-2,4-hexadiene *	0.399	1.594	0.292
60	1597	6-methyl-3,5-heptadien-2-one	1.170	2.734	0.001
61	1610	terpinen-4-ol	1.131	9.180	0.003
62	1615	1-*p*-menthen-9-al *	1.125	1.547	0.002
63	1629	γ-butyrolactone	0.599	1.140	0.080
64	1637	butanoic acid	0.582	2.443	0.220
65	1642	phenylacetaldehyde	1.305	0.397	0.001
66	1653	acetophenone	0.511	1.078	0.118
67	1679	3-methylbutanoic acid	0.726	0.671	0.083
68	1685	neral	0.419	0.936	0.346
69	1707	α-terpineol	1.412	2.242	0.000
70	1728	methionol	0.936	2.027	0.012
71	1736	geranial	0.275	0.999	0.542
72	1744	1, 2-dihydro-1,1,6-trimethylnaphthalene *	0.381	2.257	0.249
73	1776	methyl salicylate	1.080	5.854	0.001
74	1811	2, 3-dimethylbenzaldehyde	0.329	0.533	0.267
75	1826	(*E*)-β-damascenone	0.469	1.302	0.257
76	1855	hexanoic acid	1.419	3.187	0.000
77	1861	(*E*)-geranylacetone	1.123	3.081	0.004
78	1866	2-methoxyphenol	1.317	13.191	0.000
79	1885	benzyl alcohol	1.663	5.799	0.000
80	1920	phenylethyl alcohol	1.610	6.862	0.000
81	1929	benzonitrile	0.572	0.626	0.328
82	1944	β-ionone	1.373	3.552	0.000
83	1961	2-ethylhexanoic acid	0.583	1.477	0.107
84	1998	2H-pyran-2,6(3H)-dione *	0.976	1.437	0.042
85	2030	formylpyrrole	1.271	0.290	0.000
86	2078	octanoic acid	0.539	0.688	0.129
87	2098	ψ-ionone	0.470	0.970	0.414
88	2142	ψ-ionone isomer *	0.877	2.619	0.017
89	2173	eugenol	1.247	16.981	0.000
90	2184	4-ethylphenol	1.162	9.502	0.001
91	2201	2-methoxy-4-vinylphenol	1.315	3.724	0.000
92	2343	dihydroactinidiolide *	1.223	2.510	0.001
93	2400	2, 3-dihydrobenzofuran	1.287	2.406	0.000
94	2435	benzoic acid	0.259	1.054	0.436

* These compounds were annotated using Aroma Office with the NIST library.

**Table 4:** Odor characterization and odor active values (OAVs) of aroma-active compounds in tomato juices.

Peak No.	RI	Identified Compounds	Odor Description	Odor Threshold		OAVs of Tomato Juices *	
				μg/L(kg) in Water	Ref.	JNFC	JFC
2	752	dimethyl sulfide	seaweed, cooked corn	0.84	[31]	318–1202 (802)	760–2030 (1370)
9	917	3-methylbutanal	cooked, malty	0.5	[31]	119–543 (183)	225–1354 (629)
18	1085	hexanal	green, grassy	4.5	[8]	2.43–117 (37.5)	2.26–96.3 (14.2)
29	1227	2-pentylfuran	fatty, metallic	6	[29]	0.0320–0.659 (0.213)	0.0298–0.444 (0.187)
42	1343	6-methyl-5-hepten-2-one	fruity	50	[8]	0.856–7.93 (4.57)	1.37–7.37 (3.07)
43	1367	hexanol	green, leafy	200	[8]	0.004–1.94 (0.488)	0.003–0.476 (0.0670)
45	1397	(*Z*)-3-hexen-1-ol	green, leafy	3.9	[31]	0.506–166 (64.5)	0.732–65.4 (8.64)
46	1410	2-isobutylthiazole	tomato vine	3.5	[8]	0.337–16.7 (7.09)	0.312–17.6 (2.91)
52	1457	methional	boiled potato	0.43	[32]	26.8–65.3 (47.1)	54.4–125 (82.8)
53	1462	1-octen-3-ol	mushroom-like	14	[8]	0.002–0.061 (0.0250)	0.002–0021 (0.006)
58	1558	linalool	sweet, flower-like	6	[8]	0.254–3.69 (1.35)	0.245–1.14 (0.563)
64	1637	butyric acid	cheese-like	50	[30]	0.134–5.31 (0.893)	0.134–1.36 (0.366)
65	1642	phenylacetaldehyde	floral	5.2	[32]	0.415–2.71 (1.56)	1.54–6.75 (3.92)
67	1679	3-methylbutanoic acid	cheese-like	490	[32]	0.0156–0.127 (0.047)	0.0234–0.128 (0.070)
71	1736	geranial	citrus-like	32	[8]	0.0184–0.419 (0.119)	0.0483–0.194 (0.119)
73	1776	methyl salicylate	medicinal	40	[8]	0.005–0.158 (0.049)	0.00607–0.0108 (0.008)
75	1826	(*E*)-β-damascenone	sweet, floral	0.013	[32]	303–1334 (706)	338–1183 (542)
78	1866	2-methoxyphenol	medicinal	0.84	[32]	0.258–19.2 (5.88)	0.032–0.912 (0.446)
80	1920	2-phenylethanol	floral	140	[31]	0.249–2.02 (0.921)	0.055–0.385 (0.134)
82	1944	β-ionone	fruity	0.007	[8]	43.4–935 (367)	60.6–485 (140)
89	2173	eugenol	spicy, woody	6	[8]	0.155–9.58 (2.50)	0.121–0.176 (0.147)
91	2201	2-methoxy-4-vinylphenol	spicy	5.1	[31]	0.774–9.31 (2.72)	0.474–1.21 (0.731)

* OAVs indicate the minimum to maximum of the samples in each group. Numbers in parentheses represent averages.

## Data Availability

The original data used in this study are included in the article/Supplementary Material. Further inquiries can be directed to the corresponding author.

## Supplementary Materials

The following supporting information can be downloaded at: https://www.mdpi.com/article/10.3390/foods14172993/s1, Table S1: The tomato juice used in this study obtained from Japanese supermarkets; Table S2: Relative peak intensities of each VOC relative to the internal standard (1-ethyl cyclohexanol; *m*/*z* 99).

## Abbreviations

The following abbreviations are used in this manuscript:

VOC	Volatile organic compounds
JNFC	Juice not from concentrate
JFC	Juice from concentrate
DVB/CAR/PDMS	Divinylbenzene/carboxen/polysimethylsiloxane
HS-SPME-GC-MS	Headspace solid phase microextraction -gas chromatography mass spectrometry
PCA	Principal component analysis
OPLS-DA	Orthogonal partial least squares discriminant analysis
GC-MS/O	GC-MS/olfactometry
SAFE	Solvent-assisted flavor evaporation
